# Commentary: Novel strategies and new tools to curtail the health effects of pesticides

**DOI:** 10.1186/s12940-021-00773-4

**Published:** 2021-08-03

**Authors:** Charles Benbrook, Melissa J. Perry, Fiorella Belpoggi, Philip J. Landrigan, Michelle Perro, Daniele Mandrioli, Michael N. Antoniou, Paul Winchester, Robin Mesnage

**Affiliations:** 1 Heartland Health Research Alliance, 10526 SE Vashon Vista Drive, Port Orchard, WA 98367 USA; 2 Department of Environmental and Occupational Health, George Washington University, Washington, DC USA; 3 Ramazzini Institute, Bologna, Italy; 4 Schiller Institute for Integrated Science and Society, Boston College, Newton, MA 02467 USA; 5 Gordon Medical Associates, San Rafael, CA 94901 USA; 6 Gene Expression and Therapy Group, Department of Medical and Molecular Genetics, King’s College London, Faculty of Life Sciences and Medicine, Guy’s Hospital, London, UK; 7 School of Medicine, Department of Pediatrics, Indiana University, Indianapolis, IN USA

## Abstract

**Background:**

Flaws in the science supporting pesticide risk assessment and regulation stand in the way of progress in mitigating the human health impacts of pesticides. Critical problems include the scope of regulatory testing protocols, the near-total focus on pure active ingredients rather than formulated products, lack of publicly accessible information on co-formulants, excessive reliance on industry-supported studies coupled with reticence to incorporate published results in the risk assessment process, and failure to take advantage of new scientific opportunities and advances, e.g. biomonitoring and “omics” technologies.

**Recommended Actions:**

Problems in pesticide risk assessment are identified and linked to study design, data, and methodological shortcomings. Steps and strategies are presented that have potential to deepen scientific knowledge of pesticide toxicity, exposures, and risks.

We propose four solutions:

(1) End near-sole reliance in regulatory decision-making on industry-supported studies by supporting and relying more heavily on independent science, especially for core toxicology studies. The cost of conducting core toxicology studies at labs not affiliated with or funded directly by pesticide registrants should be covered via fees paid by manufacturers to public agencies.

(2) Regulators should place more weight on mechanistic data and low-dose studies within the range of contemporary exposures.

(3) Regulators, public health agencies, and funders should increase the share of exposure-assessment resources that produce direct measures of concentrations in bodily fluids and tissues. Human biomonitoring is vital in order to quickly identify rising exposures among vulnerable populations including applicators, pregnant women, and children.

(4) Scientific tools across disciplines can accelerate progress in risk assessments if integrated more effectively. New genetic and metabolomic markers of adverse health impacts and heritable epigenetic impacts are emerging and should be included more routinely in risk assessment to effectively prevent disease.

**Conclusions:**

Preventing adverse public health outcomes triggered or made worse by exposure to pesticides will require changes in policy and risk assessment procedures, more science free of industry influence, and innovative strategies that blend traditional methods with new tools and mechanistic insights.

## Background

Today’s near-total dependence on pesticides for weed, insect, and pathogen control on conventionally managed farms is likely unsustainable. Steadily rising pesticide expenditures reduce net returns to farmers. Heavy pesticide use imposes collateral damage on farmers and workers, the environment, and public health that can become unacceptable as a result of direct costs; fines, penalties, or more costly insurance; regulation; social pressure or local controls. Examples of such damage include crop and tree losses due to dicamba and other herbicide drift [1, 2], reduced biodiversity [3–6], diminished ecosystem services [7, 8], pollinator decline [8, 9], acute poisonings [9], the emergence and spread of resistant weeds [10] and insects [11], loss of vital drugs needed to treat human infections [12], and water contamination [13, 14].

Although the human health impacts stemming from exposure to pesticides are not fully known or understood, an increasing number of studies describe adverse effects following acute occupational exposures [9], heightened risk of cancer [15–18], and residential proximity to farms heavily reliant on pesticides [19, 20].

In the US, European Union, and other developed countries, pesticides undergo an evaluation of their environmental and human health effects before they are placed on the market. However, current regulatory systems have often failed to detect and/or mitigate some of the health effects triggered by pesticides. Examples include damage to the neurological system and brain from chlorpyrifos and other organophosphates [19, 21], the impact of exposures to glyphosate-based herbicides on non-Hodgkin lymphoma [15, 22, 23], and adverse reproductive impacts of multiple pesticides [24, 25]. These failures to detect and prevent adverse health outcomes arise from systemic shortcomings in how regulators, industry and public health professionals assess pesticide risks and strive to mitigate them. Because of these shortcomings, problems can linger for years, as in the case of:

- The role paraquat has played in the etiology of Parkinson’s disease [26–28],
- Impacts of prenatal exposures to glyphosate-based herbicide on preterm births [24, 29], the gut microbiome [30], and non-alcoholic fatty liver disease [31, 32], and
- Exposures leading to autism and autoimmune diseases [20, 33].

In some of the cases cited above, substantial evidence of toxicity and significant human risk had been published many years, or even decades before the EPA or other regulators acted to restrict use and exposures [34]. Other shortcomings stem from the limited set of tools regulators utilize to quantify and mitigate risks arising from how pesticides are actually used in the real world, in contrast to how pesticides are supposed to be used in accord with label directions [35].

The science supporting pesticide risk assessment and regulation has been reviewed critically in recent years [36, 37]. After providing an overview of the most consequential problems in how pesticides are used and risks are managed in the US and other countries, we propose strategies to:

- Deepen scientific knowledge of adverse pesticide impacts,
- Build trust in regulatory risk assessments, and
- Broaden support for steps to reduce overall reliance on pesticides, and especially use of high-risk pesticides.

The paper focuses predominately on shortcomings and challenges confronting pesticide risk assessors and regulators in the US and the European Union. In general, these countries follow guidelines and testing principles from the Organization for Economic Co-operation and Development (OECD). Our recommendations are likely to apply to other OECD countries, and to developing countries with regulations that draw upon OECD standards, e.g. members of the Economic Community of West African States (ECOWAS) [38]. Progress in the sciences and policies governing pesticide use has potential to support reductions in the adverse public health impact of pesticide use in other parts of the world where the need for significant reductions in the frequency of high-exposure episodes is more acute.

## Flaws in the science supporting pesticide risk assessment and regulation

### Formulation chemistry matters

Pesticide formulation chemistry is a complex interplay of different ingredients at varying concentrations. Formulations directly impact product effectiveness, environmental fate, and risk profiles. Applications via specialized spray equipment, as part of regional pest management systems, or on particular soils can require different formulations for optimal efficacy [39]. Some formulations allow the mixing of pesticides with liquid fertilizers, so one pass through a field can accomplish multiple tasks.

So-called “inert ingredients” in formulated herbicides enhance adhesion and allow herbicides to move more quickly through the epidermis of weeds. They help reduce drift and prevent clogged nozzles. Some surfactants also markedly enhance toxicity to non-target organisms [40] and human-health risks [41]. Applicator exposure episodes leading to glyphosate doses high enough to trigger ocular damage [42], skin irritation [43], non-Hodgkin lymphoma [15], and gastrointestinal disorders [44, 45] were not due to glyphosate alone, but to the glyphosate plus surfactants in formulated commercial products (e.g. MON 0818 for Roundup MON 2139) [41, 46, 47].

But regulatory reviews largely ignore the impacts of co-formulants. In the US, nearly all pesticide risk assessments are based on studies done on the nearly pure “active ingredients” — “active” in the sense that they are responsible for the desired impact on weeds, insects, plant diseases, rodents, or other pests. But when applied in the field, nearly all pesticides are complex mixtures with multiple adjuvants and surfactants and an active ingredient. Many pesticide products contain more than one active ingredient, and some up to three, as in the case of Expert® herbicide that includes a mix of S-metolachlor, atrazine and glyphosate (EPA Reg. No. 100–1161) [48].

In the past decade, published data have confirmed what regulators and industry have known for years: substantial differences exist in the risk profile of pesticide active ingredients in contrast to the formulated products containing them. These differences alter environmental fate, metabolism, and excretion [41]. This is clearly the case with the world’s leading herbicide glyphosate in vivo in rats [47, 49] or in vitro in human cells [46], and for the most widely used family of insecticides (neonicotinoids) [50, 51].

Moreover, current US and EU law allows pesticide registrants to claim that the formula of commercial products are confidential business information. Hence, there is often no way to know what is in a given end-use pesticide. In the case of glyphosate, hundreds of products containing glyphosate are available in the US. These products have different names, although they can have the same, or very similar composition. They are often marketed under alternate names when sold in another country. Herbicide manufacturers use a trade name (e.g. Roundup) and unique formulation number (e.g. MON 2139) to accurately identify and link a product back to its confidential statement of formula. But the internal formulation number and its contents and concentrations are rarely disclosed [41]. Farmers and scientists cannot be expected to understand and manage efficacy issues and pesticide risks without detailed and specific information on what is in the products actually applied.

### Occupational exposures are an afterthought

Applicators, farmworkers and those occupationally exposed, or living near treated fields, face heightened risks compared to the general public, as is apparent in most pesticide human health risk assessments completed by the EPA (e.g. glyphosate [52], paraquat [53], or chlorpyrifos [54]). There are many causes of this emerging environmental justice issue:

- Much less rigorous focus on the exposure data available to quantify occupational exposures, especially in contrast to dietary exposures,
- Highly questionable data supporting estimates of dermal absorption rates derived predominately from studies using pure active ingredients rather than formulated products,
- The assumptions that: (a) all required personal protective equipment will be appropriate to achieve the hoped-for reduction in exposure, in good working order, and properly utilized, and (b) all requirements, precautions, and warnings will be adhered to, and
- The generally higher risks deemed acceptable by regulators when farmworkers and applicators are exposed to pesticides, in contrast to the general public.

Applicator, farm worker, and other bystander and occupational exposures and risks are presumptively managed by changing use rates or application methods, by imposing incrementally more Personal Protective Equipment (PPE), via engineering controls, or other measures. Requiring more PPE may work on paper, but often falls short in practice [33]. Applicators of high-risk pesticides are often unable or not willing to comply with all existing PPE and use directions requirements, or do so inappropriately, undercutting the reductions in exposures projected by regulators [55].

The science supporting current applicator and mixer-loader exposure and risk assessments is plagued by poor data (e.g. exposure estimates from models estimating the impacts of various combinations of PPE, rather than direct measurements) and methodological flaws (incorrect dermal absorption rates, studies based on pure active ingredients rather than formulations). Actual exposures derived from field-level biomonitoring studies are often far higher than what the EPA has estimated [56]. This is especially true in the case of applicators spraying pesticides with handheld and backpack sprayers [57, 58]. A person applying one pound of pesticide active ingredient in a 6-h workday using handheld equipment will almost always experience tenfold to 100-fold higher exposure than the operator of a modern pesticide sprayer who sits in a glass-steel cab and sprays 1,000 lb of pesticide in a day [59].

Deficiencies and gaps in the data supporting applicator and occupational risk assessment give rise to systemic underestimation of worker exposures and risk. Estimates of typical rates of dermal absorption for many pesticides are too low [60, 61]. The pesticide industry prefers to use, and regulators have unfortunately accepted, non-viable skin-based penetration assays that underestimate actual rates [62]. Additionally for most pesticides, dermal absorption rates are derived from studies of pure active ingredient, while in the field, exposures always occur to formulated pesticides containing surfactants, many of which enhance dermal penetration [63]. Pesticide manufacturers are aware of this, but have generally not taken the actions needed to sharpen the accuracy of applicator and worker risk assessments. A common explanation from registrants is that they comply with all requirements imposed on them by regulators.

The EPA and EU regulators have begun asking registrants to carry out exposure-route specific toxicity tests to set Points of Departure (POD) for risk assessments. A POD is typically the Lowest Observed Adverse Effect Level (LOAEL) in an animal assay. Regulators then try to assure that exposure levels in a day will be at least 100-fold or more lower than the estimated POD. But when the EPA accepts a toxicity study done in an animal model that entails treatments with a pure active ingredient rather than the formulated products, unrealistically high PODs can result, further separating applicator and occupational risk assessments from real-world conditions. Similar sources of uncertainty arise in the way worker risks are evaluated in other countries. Estimates are often based solely or largely on data generated and submitted by registrants.

### Evaluating real-life exposure scenarios

Most pesticide toxicology data requirements rely on outdated testing methods that either originated from or have been influenced by guidelines issued by the OECD [64]. The core toxicology tests required by the EPA, EU, and most other regulatory authorities focus almost exclusively on pure active ingredients. Most methods dose adult animals up to a Maximum Tolerated Dose (MTDs). This provides vital information for hazard classification. However, pesticide doses to which human populations are exposed in the environment (e.g. via diet) are much lower, with the possible exception of some applicator exposure episodes entailing use of handheld or backpack sprayers and little or no PPE.

Current risk assessment methods are based, for the most part, on the assumption that the severity of toxic effects at low environmental doses can be approximated using linear extrapolations from effects measured at higher doses. A large number of studies have now demonstrated that this is not true for hormonal effects [65, 66].

Likewise, conventional pesticide risk assessment does not adequately consider the impacts of exposures in early fetal development when the timing of exposure may be as important, or even more important, than dose [67]. Moreover, OECD guideline studies are not designed or able to detect some cancers with long latency periods nor neural decline, and other diseases that typically become evident later in life [68]. OECD testing protocols are especially limited in their ability to detect adverse health impacts stemming from low-level chronic exposures, and from periodic high-level exposures among people mixing, loading and applying pesticides, or working or living in or near heavily treated fields.

New strategies for assessing low-dose, non-linear and early developmental toxicity have been developed. These include the use of high-throughput technologies known as ‘omics’ methods (metabolomics, transcriptomics, genomics, metagenomics, or even epigenomics). These methods allow the monitoring of multiple biomarkers and can identify metabolic perturbations that typically remain undetected despite full compliance with current regulatory test requirements (Table 1). This was the case in a recent study of a mixture of six pesticides frequently detected in foodstuffs [69].

**Table 1:** Multiple Omic layers are available to serve both as an exposure and health outcome biomarkers. Other omics methods such as glycomics or ncRNomics have been used to understand human health but they have not been extensively used in toxicology

Omics	Identified health hazards	Identified harmful exposure	Predicted health outcomes
Transcriptomics *RNA transcripts*	▪ *Genotoxicity:* In vitro transcriptional signatures classify agents as genotoxic or non-genotoxic in human lymphoblastoid TK6 cells [70] ▪ *Endocrine disruption:* A Gene Expression Biomarker Accurately Predicts Estrogen Receptor α Modulation in breast cancer MCF-7 cells [71] ▪ *Toxicity point of departure:* Rat liver transcriptional signatures in a short-term exposure can estimate a toxicity point of departure for longer-term effects [72]	▪ *Tobacco smoking:* Smoking-related gene expression signatures can be detected in whole-blood [73] ▪ *Metformin use:* Transcriptome signatures identify metformin use and discriminate between metformin responders and non-responders, explaining variance in therapeutic efficacy [74]	▪ Nonalcoholic fatty liver disease: *Hepatic transcriptome signatures predict disease progression in patients with varying degrees of liver diseases* [75] ▪ *Cancer prognostic*. A gene expression signature supports physicians' treatment decisions in a population with early breast cancer [76] ▪ *Tumor tissue origin*: Transcriptional signatures can help identify the tissue of origin for metastatic cancers [77]
Metabolomics *small molecules*	▪ *Gut microbiome alterations*: Caecum metabolites levels reflect shikimate pathway inhibition by the herbicide glyphosate in the rat gut [30] ▪ *Improved toxicity predictions*: Results of 90-day rat toxicity studies can be predicted using metabolome data of 28 day studies [78] ▪ In vivo* toxicity from *in vitro* assays*: Liver toxicity mechanisms can be predicted from metabolite profiles of HepG2 cells [79]	*▪ Pesticides*: An exposure to complex pesticide mixtures induces modifications of metabolic fingerprints in pregnant women according to the intensity of agricultural cereal activities [80] ▪ *Heavy metals*: Urinary metabolite profiles could reflect arsenic internal dose-related biochemical alterations [81] ▪ *DDE and HCB*: Circulating levels of 16 metabolites related to lipid metabolism reflect p,p'-DDE and HCB exposure in humans [82]	▪ *All-cause mortality:* A set of 14 metabolites act as predictors of long-term mortality in the circulation of 44,168 individuals [83] ▪ *Breast cancer:* Metabolites show potential as biomarkers for early diagnosis of breast cancer, predicting tumor size and hormone receptor expression [84] ▪ *Neurodegeneration:* Blood lipids identify antecedent memory impairment and can act as diagnostic tools for early neurodegeneration of preclinical Alzheimer's disease [85]
Genomics *genome sequence*	▪ *Susceptibility to chemical toxicity:* Response to chemical exposure in genetically heterogeneous zebrafish can help elucidate gene-environment interactions [86]	Not applicable	▪ *Variations in disease risks:* Genome-wide polygenic scores can identify individuals at high risk for five common diseases [87]
Metagenomics *microbial communities*	▪ *Microbiome drug metabolism*. Interpersonal differences in drug metabolism can be identified by high-throughput genetic analyses of gut microbiomes [88] ▪ *Melamine toxicity:* Melamine-induced renal toxicity depends on the exact composition and metabolic activities of the gut microbiota [89]	▪ *Personalised responses to diet:* The composition of the gut microbiome predicts personalised glycemic responses to food [90] ▪ *Heavy metals:* Rats exposed daily to arsenic, cadmium, cobalt, chromium, nickel display changes to microbiota composition which can help identifying exposures to specific heavy metals [91]	▪ *Cardiometabolic health:* Gut microbiome composition is predictive for cardiometabolic blood markers in 1,098 deeply phenotyped individuals [92] ▪ *Cirrhosis:* Gut microbiome species can be a non-invasive diagnostic test for cirrhosis [93]
Epigenomics *DNA modifications and chromatin structure*	▪ *Transgenerational inheritance*: The study of epigenomes and chromatin accessibility informed on transgenerational inheritance after ancestral perinatal obesogen exposure [94] ▪ *Tissue susceptibility*: Epigenetic marks determine differential tissue susceptibility to tumorigenesis induced by 1,3-butadiene [95]	▪ *Maternal smoking*: Epigenetic changes in response to maternal smoking in pregnancy persist into later childhood [96] ▪ *Air pollution:* DNA methylation reprogramming after prenatal exposure to air pollution was associated with markers of cardiovascular risk in childhood [97]	▪ *Death risk*: Site-specific blood DNA methylation sites predict death risk in a longitudinal study of 12,300 individuals [98] ▪ *Colorectal cancer*: A panel of DNA methylation biomarkers in peripheral blood could predict colorectal cancer susceptibility [99]
Proteomics *Proteins and peptides*	▪ *Sex differences*: Proteomics was used to detect sex-related differences in effects of toxicants [100] ▪ *Drug toxicity prediction*: proteomic signatures associate with hepatocellular steatosis in rats [101]	▪ *Radiation injury:* comparative proteomic allowed the discovery of new radiation biomarkers [102] ▪ *Heavy metals:* A panel of six proteins was proposed to serve as marker of occupational exposures to arsenic, cadmium, and lead [103]	▪ *Health and life span*: A 76-protein proteomic age signature predicted chronic diseases and all-cause mortality in 997 individuals between 21 and 102 years of age [104] ▪ *Cancer*: Differentially expressed serum proteins could be used for early diagnosis and pathogenic investigation of Non-Hodgkin lymphoma [105]

While standard histopathology and serum biochemistry measures revealed few effects, the combination of high-throughput omics revealed the activation of stress-response pathways. Metabolomics or RNA sequencing of sensitive tissues or in urine or blood can also be deployed to identify biomarkers and predict the risk of long-term health effects from pesticide exposures (Table 1). For instance, exposure to complex pesticide mixtures induces specific modifications of metabolism in pregnant women [80]. The profiling of small RNA in urine samples from individuals who ingested high doses of paraquat or glyphosate identified acute kidney injury [106]. In another study, small RNAs in urine were associated with organophosphate metabolites in farmworkers [107]. It was even hypothesized that small RNA could provide a link between exposure to pesticides and the development of Parkinson's disease [108]. Novel mechanistic assays have also been developed to allow the identification of key characteristics of human carcinogens [109].

### Reliance in risk assessments on industry-sponsored studies versus published studies by scientist not funded by or affiliated with pesticide manufacturers

The majority of studies considered by pesticide regulators are undertaken in compliance with Good Laboratory Practice (GLP) guidelines [110]. GLPs are intended to ensure consistency and reliability in laboratory testing. Studies that do not adhere to stated GLP guidelines, but are otherwise sound, are typically not considered or are given little weight in “weight of the evidence” judgements. This bias against published, non-GLP studies excludes consideration of mechanistic insights from genomic analyses (see Table 1 for an overview of emerging genomic tools that can support pesticide risk assessment) [111, 112].

The EPA’s assessment of glyphosate oncogenicity is a good example of the pitfalls inherent in relying on industry-supported, GLP-compliant toxicology studies [59, 113]. The EPA determined in 2015 that glyphosate is “not likely to be carcinogenic to humans.” This finding contrasted with the “probably carcinogenic to humans” Group 2A classification issued by the International Agency for Research on Cancer (IARC), also in 2015 [23, 114]. Why the difference?

In the most recent cycle of reregistration of glyphosate-based herbicides in both the US and EU, regulators relied mostly on industry-conducted, GLP-compliant glyphosate genotoxicity assays, of which about 1% reported a positive result. Over one-half of these assays were bacterial mutation assays which are known to have a limited sensitivity [115]. The IARC glyphosate Working Group placed heavy weight on the nearly three-quarters of published glyphosate genotoxicity studies that reported one or more positive assays [59, 114].

Industry genotoxicity studies reporting no evidence of genotoxicity were mostly done in the 1980s and 1990s, while most positive published studies were done since 2000 and were conducted with generally more sensitive assay systems. Over 90% of the glyphosate genotoxicity assays conducted since completion of the IARC glyphosate monograph have reported one or more positive assays [116]. IARC placed heavy weight on in vivo studies with formulated glyphosate-based herbicides (GBHs), including three studies in human populations exposed as a result of aerial spraying that reported evidence of genotoxicity in exposed people compared to people living nearby who were not exposed.

Other examples of substantial gaps between the findings of industry-sponsored GLP studies and published studies conducted by university-based scientists include studies on the endocrine disrupting effects of endosulfan [117, 118], the reproductive impacts of the synthetic pyrethroid insecticides [39, 119], and the developmental toxicity of chlorpyrifos [68, 120].

### Multiple concurrent exposures and vulnerable populations

Another shortcoming with traditional toxicology testing — which examines each pesticide in isolation — is that no one is exposed to just one pesticide, nor are people exposed only to pesticides [121]. Biomonitoring data generated in the US by the Centers for Disease Control and Prevention demonstrate that in recent years more than 90% of Americans have a few to several pesticides and/or pesticide metabolites in their body on any given day [122, 123], as well as several other chemicals, e.g. metals, furans, polycyclic aromatic hydrocarbons, plasticizers such as phthalates and bisphenols, or brominated and fluorinated chemicals [124]. Some can accumulate in a variety of human tissues, others can be found in breast milk [125] and pass via the placenta into the fetus [126].

Pesticide biomonitoring data has been published reporting the frequency and levels of pesticide analytes in human urine [124, 127–129]. Expanded EU investments in biomonitoring will produce additional data to support refined exposure and risk assessments and epidemiological analyses [130].

Further complications in pesticide risk assessment arise as a result of the well-known fact that not all members of exposed populations are equally vulnerable to pesticides. A variety of genetic polymorphisms, health conditions, and drug therapies render segments of the population more vulnerable following exposure to certain pesticides [131]. Since the enzyme paraoxonase 1 hydrolyzes most organophosphorus insecticides in plasma and the liver, its activity, or lack thereof in some people, modulates organophosphate toxicity by an order of magnitude, and for some people, up to two orders of magnitude [132, 133]. Low paraoxonase status increases susceptibility to disruption in children’s neurobehavioral development [134, 135]. Maternal levels of chlorpyrifos coupled with low maternal PON1 activity were associated with a significant reduction in head circumference in their offspring [136].

Dietary factors such as malnutrition and vitamin deficiency can enhance the toxic effects of some pesticides. A recent animal study suggested that chronic vitamin deficiency can influence the toxicity of a pesticide mixture [137]. Another study showed that neonicotinoids and nutritional stress can act synergistically to reduce survival of honey bees [138]. Epidemiological studies clearly indicate that agricultural workers exposed to pesticides are subject to damaging oxidative stress and this stress can activate antioxidant responses [139]. Further studies are needed to determine whether and to what extent a person’s nutritional status can influence the development of pathologies after pesticide exposures.

People living in farming areas, in buildings sprayed for insects, or near golf courses experience added exposures. Multiple pesticides with endocrine disrupting activity have been found in public playgrounds and schoolyards in agricultural areas [140]. People mixing and loading pesticides and applying them are by far the most heavily exposed [141], especially those using handheld, backpack, or ATV-mounted sprayers [37, 57, 116], yet no product labels have warnings addressed to more highly exposed occupational users, nor pregnant women or individuals currently receiving chemotherapy. In the US, many plaintiffs in the Roundup-non-Hodgkin litigation continued to spray Roundup post NHL diagnosis and between rounds of chemotherapy and/or stem cell transplants. When asked why by counsel, some testified that no one alerted them to the need to avoid future exposures to possibly cancer-causing chemicals.

Current EPA and EU pesticide risk assessment protocols and label warnings sometimes touch upon such differences, but do so inadequately. The warnings and exposure-reduction provisions on pesticide labels do not generally take into account nor address the heightened vulnerability of certain population cohorts. Labels on paraquat products fail to warn of neurotoxicity and do not mention the possible link between exposures and Parkinson’s disease [35]. Nearly all labels also fail to alert applicators spraying pesticides dozens of times annually that some health risks rise with repeated exposures. Nor do labels emphasize, or even mention the need for discipline to avoid high-risk scenarios caused by weather conditions, a leaky hose, or spray patterns. Rectifying these shortcomings warrants a global response across regulatory authorities, better data from specific exposure-scenario research, and policy reforms.

## Four Solutions to Curtail the Adverse Health Effects of Pesticides

### Independent science in support of pesticide risk assessment and regulation is sorely needed

Toxicology studies that underpin pesticide risk assessment and standard setting should largely be undertaken and analyzed by independent scientists. Currently in the US and in most of the world, almost all of the data used to regulate pesticides are derived from unpublished studies conducted by pesticide registrants, or companies working under contract for pesticide manufacturers [142]. Their job is to produce study results that support — and defend — existing pesticide tolerances, uses and registrations, and for the most part that is what they do.

As long as existing pesticide testing protocols remain the basis for regulation and public health protection, little progress is likely in mitigating adverse public health impacts stemming from pesticide use. The gaps between the risks that regulators recognize and strive to mitigate, and risks in the real world are sizable and greatest among those who are heavily exposed and/or more highly vulnerable.

The primary toxicology data packages supporting initial registration of new active ingredients are typically carried out in the US or EU and submitted for evaluation to the EPA and European Food Standards Agency (EFSA). The same core studies are usually submitted to multiple regulatory authorities around the world. To diversify and enhance the quality of the science base supporting pesticide risk assessments, the US EPA and EFSA should agree upon a process whereby registrants must provide sufficient funding via registration-process fees for the EPA or EFSA to sponsor a set of core toxicology studies at a laboratory with appropriate research capability that is not affiliated with the industry. Another solution which could be implemented at essentially no cost is the requirement for industry-supported studies to be registered in advance in a publicly accessible database like clinical trials for drug safety, in order to prevent companies from halting and never disclosing studies that seem to be generating unwelcomed results.

Adoption of these new approaches to enhance the quality of core pesticide toxicology studies won’t materially change the cost of studies supporting pesticide risk assessment, unless a registrant decides to also fund and/or conduct comparable studies inhouse. In either case, this approach should, over time, enhance the quality and reliability of studies supporting key regulatory decisions, and overtime, enhance public trust in the science supporting regulatory decisions.

### Invest in direct measures of exposure

To determine which pesticides warrant stricter regulation, more biomonitoring data are essential. For example in the Midwestern US, the spread of glyphosate-resistant weeds is driving upward the use of 2,4-D, dicamba, and glufosinate [143, 144]. Tracking the public health impacts of rising herbicide use in this region requires accurate measures of changes in exposure. Such measures can best be obtained via biomonitoring.

Efforts are underway in many countries to harmonize and aggregate human biomonitoring data to better identify vulnerable or highly exposed populations. The European Joint Program HBM4EU [128] shows promise in improving both the volume and quality of biomonitoring data across the EU. Biomonitoring human exposure to glyphosate co-formulants has been designated as a priority by HMB4EU [145]. A method to do so was recently created and applied to the study of the urinary excretion of Roundup MON 52,276 co-formulants in exposed rats [146]. In the US, the CDC’s National Health and Nutrition Examination Survey [122] has recently developed new methods to test the most heavily applied herbicide (glyphosate [147]) and the insecticides people are most frequently exposed to via diet (the neonicotinoids [148]).

### Place more weight on mechanistic data

DNA damage is a worrisome consequence of pesticide exposure and can occur via multiple mechanisms including oxidative stress, chromosomal strand breaks, and formation of adducts. A pesticide’s ability to damage DNA directly or indirectly is correlated with its carcinogenicity [149], ability to trigger developmental anomalies [150], and impacts on endocrine system function [151].

Prevention of oxidative stress and DNA damage should be key objectives of pesticide regulation [152, 153]. Yet the toxicological studies currently supporting pesticide registrations are not always capable of identifying the full range of metabolic and genetic mechanisms leading to, causing, or exacerbating DNA damage [154]. New methods that can identify genotoxic compounds with high accuracy and characterize their oxidative stress related mode, or modes of action are already available [155]. While bacterial mutation assays (Ames tests) mentioned above were able to detect genotoxic potential in 46% of a set of test compounds, new assays did so with 95% accuracy [156]. This new generation of assays were recently used to compare genotoxicity profiles of glyphosate and three formulations [109]. This work revealed that two Roundup herbicides but not glyphosate activated oxidative stress and led to misfolded proteins, two key characteristics of human carcinogens [149].

Metabolomics is a cutting-edge technology that has much to offer in emerging pesticide risk assessment methods [157]. New findings suggest exposures to the pesticides atrazine, diazinon, glyphosate-based herbicides, and trichlorfon cause sex-specific shifts in gut microbiota, which have been linked to many adverse effects in children and young adults [158–160].

For over a decade, metabolomics has been considered sufficiently mature to provide critical information for use in clinical medicine [161]. Metabolomics has, moreover, contributed to real-time diagnostics and integrative patient modeling during surgeries [162]. But guidelines for best practice and GLPs, and reporting standards for metabolomic results in chemical and pesticide risk assessment have been proposed only recently [163]. More funding is needed to pick up the pace in applications of metabolomics in regulation in order to buttress the ability of pesticide risk assessments to identify — and prevent — adverse public health outcomes.

It is encouraging that molecular profiling tools are increasingly incorporated in novel experimental systems [30, 157, 164, 165]. Such systems can detect subtle impacts triggered by chemical exposures that start the progression to disease. In recent studies, metabolomics has provided useful insights into the mechanisms leading to toxic effects of individual pesticides, and mixtures of pesticides at doses far below those triggering observable, adverse effects using standard histopathology and biochemistry methods [166].

### Knit scientific tools together to accelerate progress

Novel approaches are needed to integrate genetics and genomics, toxicology, clinical science and epidemiology. One promising strategy is applying metabolomics in search of markers of epigenetic disruption in gene expression patterns in blood, bone marrow, and gut microbiomes. Unlike typical high-dose regulatory studies in mice and rats, metabolomic assays are being carried out using dose ranges that overlap current exposure levels among exposed human populations [69, 157].

In vivo study designs need to evolve in order to assess different end-points and explore and apply new mechanistic insights (e.g. how oxidative stress can promote fatty liver disease [167] or reproductive anomalies [168]). Progress will allow reductions in the number and diversity of animal studies, with potential to cut costs and promote more rapid progress in linking specific exposures to identified endpoints of concern [169]. Such innovation will be complemented by in vitro [170] and computational approaches to predict adverse outcomes [171].

Novel integration of mechanistic insights and traditional toxicology are among the goals of the Global Glyphosate Study [172]. The pilot phase of the study suggested that a glyphosate-based herbicide at the US EPA chronic reference dose altered sexual development in a rat study, damaged DNA, and disrupted the intestinal microbiome [47, 49, 173]. A subchronic toxicity study in rats used shotgun metagenomics and metabolomics to explore the effects of glyphosate and a GBH on the gut microbiome and serum metabolome [30]. This study revealed for the first time that glyphosate and a GBH inhibit the shikimate pathway in gut microbiota, and that the blood metabolome in exposed animals was altered in ways consistent with oxidative stress.

## Conclusions

Improvements in pesticide risk assessment and regulation are particularly vital in the case of applicator and worker exposures and risks. Occupational exposures always occur to formulated products, yet most of the toxicology data supporting occupational risk assessments are derived from studies on pure active ingredients. Laws and policies prohibiting the disclosure of the surfactants and other “inert ingredient” in formulated pesticides, and their concentrations, should be amended in the interest of public health.

The surest path to achieve significant and sustained reductions in the adverse impacts of pesticides is reducing their overall use. Doing so will require more investment in public and private research and technical support for farmers to accelerate the shift toward prevention-based, biointensive Integrated Pest Management [174–176].

New approaches and strategies are essential to take full advantage of emerging mechanistic insights in the design of in vivo experiments and in the analysis of in vivo results. The creative synthesis of sequencing-based technologies with clinical assessments and epidemiology has great potential. Such innovation is needed to better identify exposures that really matter so that real-world pesticide risks can be mitigated or eliminated.

## Data Availability

The datasets used and/or analyzed during the current study are available from the corresponding author upon request.

## Abbreviations

2,4-D: 2,4-Dichlorophenoxyacetic acid (herbicide active ingredient)
ATV: All-Terrain Vehicle
CDC: United States Centers for Disease Control
CSF: Confidential Statement of Formula
EPA: United States Environmental Protection Agency
GBH: Glyphosate-Based Herbicide
GLP: Good Laboratory Practices
HBM4EU: Human Biomonitoring for the European Union
IARC: International Agency for Research on Cancer
LOAEL: Lowest Observed Adverse Effect Level
MTD: Maximum Tolerated Dose
OECD: Organisation for Economic and Cooperative Development
POD: Points of Departure
PPE: Personal Protective Equipment
